# *Pseudomonas aeruginosa* Vaccine Development: Lessons, Challenges, and Future Innovations

**DOI:** 10.3390/ijms26052012

**Published:** 2025-02-25

**Authors:** Rebeca Santamarina-Fernández, Víctor Fuentes-Valverde, Alis Silva-Rodríguez, Patricia García, Miriam Moscoso, Germán Bou

**Affiliations:** 1 Servicio de Microbiología, Instituto de Investigación Biomédica de A Coruña (INIBIC), Complexo Hospitalario Universitario de A Coruña (CHUAC), Sergas, 15006 A Coruña, Spain; rebeca.santamarina.fernandez@udc.es (R.S.-F.); victorfueval@gmail.com (V.F.-V.); alis.silva@udc.es (A.S.-R.); patricia.garcia.fernandez@sergas.es (P.G.); german.bou.arevalo@sergas.es (G.B.); 2 CIBER de Enfermedades Infecciosas (CIBERINFEC), Instituto de Salud Carlos III, 28029 Madrid, Spain; 3 Área de Medicamentos Biológicos, Agencia Española de Medicamentos y Productos Sanitarios (AEMPS), 28022 Madrid, Spain; 4 Departamento de Fisioterapia, Medicina y Ciencias Biomédicas, Universidad de A Coruña, 15006 A Coruña, Spain

**Keywords:** *Pseudomonas aeruginosa*, virulence factors, vaccines, adaptative immunity, preclinical development, antigen, antibody, cytokines, nanoparticles, clinical trials

## Abstract

*Pseudomonas aeruginosa* is an opportunistic pathogen with a multidrug-resistant profile that has become a critical threat to global public health. It is one of the main causes of severe nosocomial infections, including ventilator-associated pneumonia, chronic infections in patients with cystic fibrosis, and bloodstream infections in immunosuppressed individuals. Development of vaccines against *P. aeruginosa* is a major challenge owing to the high capacity of this bacterium to form biofilms, its wide arsenal of virulence factors (including secretion systems, lipopolysaccharides, and outer membrane proteins), and its ability to evade the host immune system. This review provides a comprehensive historical overview of vaccine development efforts targeting this pathogen, ranging from early attempts in the 1970s to recent advancements, including vaccines based on novel proteins and emerging technologies such as nanoparticles and synthetic conjugates. Despite numerous promising preclinical developments, very few candidates have progressed to clinical trials, and none have achieved final approval. This panorama highlights the significant scientific efforts undertaken and the inherent complexity of successfully developing an effective vaccine against *P. aeruginosa*.

## 1. *Pseudomonas aeruginosa*: A Clinically Relevant Pathogen

The rapid rise of antimicrobial resistance (AMR) is among the top 10 global health threats identified in the 13th General Programme of Work 2019–2023 (GPW13) [1]. Abban and Ayerakwa estimated that 4.95 million deaths worldwide are associated with AMR, warning that underdeveloped countries experience higher mortality rates [2]. There is therefore an urgent need to consider AMR a critical global public health and socio-economic problem. *P. aeruginosa* is one of the main pathogens associated with AMR-related deaths [2,3].

*P. aeruginosa* is an aerobic, nonfermenting, Gram-negative bacillus in the *Pseudomonadaceae* family and is characterized by polar flagellum-driven motility. It is found in almost all environments, ranging from water and soil to humans, and it is a pathogen of medical relevance due to its resistance mechanisms and ability to infect vulnerable populations [4]. The National Healthcare Safety Network (NHSN) and the U.S. Centers for Disease Control and Prevention (CDC) have identified *P. aeruginosa* as a prevalent pathogen in healthcare-associated infections, with particularly high incidence rates in long-term care hospitals [5]. This trend highlights the global challenge, with the American and African continents being particularly affected by this pathogen [6,7].

The opportunistic nature of *P. aeruginosa* allows it to colonize healthy skin and hospital environments. The bacterium can contaminate various surfaces and can be transmitted by healthcare workers to immunosuppressed patients such as burn victims, cancer patients, and transplant recipients [8]. This pathogen is associated with a broad range of infections, including bloodstream infections, folliculitis, otitis, urinary tract infections, and ventilator-associated pneumonia (VAP) [8,9]. VAP, the most common nosocomial infection, affects the lung parenchyma of patients who have been intubated for at least 48 h. Between 9% and 29% of such patients are affected by VAP, depending on the duration of the intubation and the underlying cause of the mechanical ventilation (e.g., surgery, disease, transplantation) [8,9,10,11,12,13]. *P. aeruginosa* is the leading cause of VAP and is also one of the most common pathogens in community-acquired and hospital-acquired pneumonias, particularly in intensive care units (ICUs) [11,12,13,14]. Patients with cystic fibrosis (CF) comprise another susceptible group frequently colonized by *P. aeruginosa*. CF is a recessive genetic disease that is most prevalent in Europe, North America and Australia. It is caused by mutations in the cystic fibrosis transmembrane conductance regulator (CFTR), which impair chloride ion transport and mucus clearance in the airways. Approximately 360 variants of the CFTR gene have been identified and classified into six main classes of CFTR mutations based on their functional effects, including defects in protein synthesis, processing or transport within the cell. The most common mutation, present in approximately 66% of CFTR alleles, is F508del, which results in CFTR misfolding and improper chloride channel function [15]. Patients with CF experience chronic inflammation of the airways and are predisposed to persistent infections by pathogens such as *P. aeruginosa* [16], which has a prevalence of approximately 24.5% in this population. The genetic adaptability of *P. aeruginosa* allows it to establish chronic infections that, together with airway inflammation, contribute to morbidity and mortality in these patients [17,18].

## 2. Pathogenesis and Virulence Factors of *P. aeruginosa*

**Figure 1:**
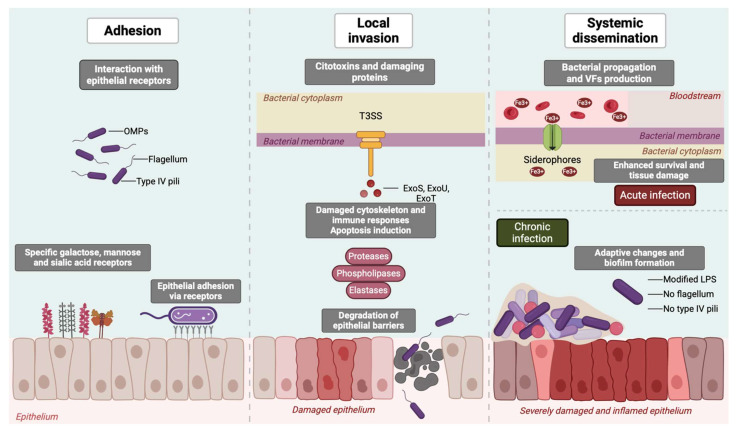
Progression of *P. aeruginosa* infection in host cells. Infection progresses through adhesion, invasion, and dissemination. Flagella, pili, and OMPs mediate adhesion and biofilm formation. The T3SS injects cytotoxins (ExoS, ExoU) that disrupt the cytoskeleton, while proteases and phospholipases degrade the epithelial barriers. Dissemination, driven by flagella, rhamnolipids, and quorum-sensing systems, promotes immune evasion and bacterial spread, leading to acute infections with severe tissue damage or chronic infections, where *P. aeruginosa* undergoes adaptive changes and downregulates VFs to enhance biofilm formation. LPS, lipopolysaccharide; OMPs, outer membrane proteins; T3SS, type III secretion system; VFs, virulence factors.

Pathogenesis in *P. aeruginosa* is mediated by a wide range of virulence factors (VFs) that facilitate adhesion, modulate host cell signaling pathways and degrade the extracellular matrix, causing severe invasive disease or persistent infections that are extremely difficult to eradicate. *P. aeruginosa* infection progresses through three key stages: adhesion/colonization, local invasion and systemic dissemination (Figure 1) [19]. In the initial adhesion phase, *P. aeruginosa* attaches to epithelial surfaces using flagella, type IV pili and outer membrane proteins (e.g., OprF), which interact with specific galactose- or mannose- or sialic-acid-containing receptors and also contribute to biofilm formation. During invasion, *P. aeruginosa* utilizes the type III secretion system (T3SS) to inject cytotoxins like ExoS and ExoU directly into host cells, which disrupt the cytoskeleton, induce apoptosis and impair immune responses. In addition, proteases, elastases (LasB) and phospholipases degrade epithelial barriers, including the glycocalyx, allowing deeper tissue penetration. During the dissemination phase, the bacterium utilizes its flagellum and rhamnolipids to spread, while quorum-sensing (QS) mechanisms coordinate VFs production. QS systems, which regulate gene expression in a cell-density-dependent manner (e.g., AHLs, DKPs, LuxR, PQS, HHQ), are a clear example of the intricate regulatory networks that connect the pathogenic mechanisms of this bacterium [20,21]. Moreover, siderophores like pyoverdine enable iron acquisition, promoting bacterial survival in the bloodstream and proliferation within the host, leading to extensive tissue damage, characteristic of acute infections. However, in chronic infections, *P. aeruginosa* undergoes adaptive changes (loss of flagella and pili, alterations in LPS, increased biofilm formation, etc.) to persist in the host environment. These adaptations allow the bacterium to adhere to the respiratory epithelium, avoid immune recognition and contribute to a sustained inflammatory state [19].

**Figure 2:**
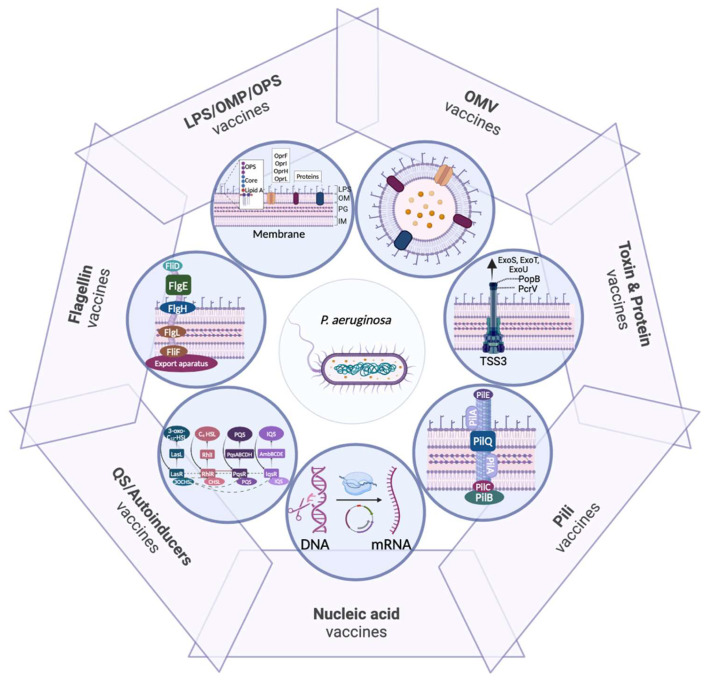
Major virulence factors as targets for vaccine development against *P. aeruginosa*. IM, inner membrane; LPS, lipopolysaccharides; OM, outer membrane; OMP, outer membrane protein; OMV, outer membrane vesicle; OPS, O-antigen; PG, peptidoglycan; QS, quorum sensing; T3SS, type III secretory system.

Among the major VFs considered as potential targets for vaccine development against *P. aeruginosa* are (Figure 2) the following.

Lipopolysaccharide (LPS) is a key component of the outer membrane (OM) and is composed of lipid A, a core region, and the O-antigen (OPS) [22]. The OPS can produce two distinct antigens: the common polysaccharide antigen (CPA) and the variable O-specific antigen (OSA), which is used for serotyping [23]. LPS plays a crucial role in interactions with host receptors, evasion of host defense mechanisms, biofilm formation and antimicrobial resistance. Specifically, lipid A has endotoxic properties that induce inflammation, stimulate the production of reactive oxygen species (ROS) and contribute to tissue damage during infections [23].

Outer membrane proteins (OMPs), such as OprF, maintain membrane integrity, regulate membrane permeability, and facilitate the acquisition of ions and nutrients. These proteins can also participate in biofilm formation and the secretion of other VFs. Additional porins, such as OprH, OprI, OprD, OprG, and OprL, contribute to antibiotic resistance, molecular transport, adhesion and membrane biogenesis [22].

Biofilm formation is a well-known characteristic of *P. aeruginosa*. An increase in c-di-GMP levels in response to environmental factors promotes secretion of adhesive molecules and matrix components, enabling the bacteria to adhere to surfaces and to each other; the bacteria then adapt physiologically until detachment of the biofilm is activated by various metabolic or environmental factors [24]. The biofilm matrix is formed by exopolysaccharides (e.g., alginate, levan, PsI, and PeI), extracellular DNA (eDNA), other polysaccharides and various proteins that act as adhesives and stabilizers, protect bacteria against external threats and impair antibiotic penetration [24,25]. Studies have demonstrated that low concentrations of aminoglycosides can induce biofilm formation [26], while alginate overproduction enhances resistance by limiting antibiotic penetration into the biofilm [27].

The type III secretion system (T3SS) is a membrane-associated system that assembles an injection complex, commonly known as the injectisome, which excretes proteins from the bacterial cytosol into the extracellular environment. The classical effector proteins are ExoS, ExoT, ExoU, and ExoY, and other molecules such as PemA/PemB and the flagellar filament. These factors disrupt immune responses, inhibit DNA synthesis and induce cell death [28,29], leading to epithelial damage and promoting bacterial persistence in the lung [22]. The type VI secretion system (T6SS) also plays a role in virulence by targeting the host microbiota and facilitating epithelial cell invasion [30].

The polar flagellum enables *P. aeruginosa* to swim in aqueous or low-viscosity environments, and it also plays an important role in chemotaxis and adhesion to mucosal surfaces, such as the pulmonary epithelium, which is protected by a thick layer of mucus [31,32].

Type IV pili are flexible, retractile appendages involved in biofilm formation, contractile motility and adherence. They are composed of pilin proteins that participate in pilus assembly (major pilins) and can have additional roles in DNA uptake and aggregation (minor pilins) [31,33].

Other secreted VFs include exotoxins and proteolytic enzymes such as elastases and alkaline protease (AprA). Exotoxin A (ETA), which is secreted via the type II secretion system (T2SS), binds to host cells and is internalized in clathrin-coated vesicles, where it induces apoptosis and necrosis and modulates inflammatory responses. Pyocyanin generates oxidative stress by increasing ROS levels, leading to cell damage and reduced lung function. Finally, the iron acquisition systems in *P. aeruginosa* include siderophores, such as pyoverdine, pyochelin and yersiniabactin, among others [34]. Overexpression of these systems is often associated with hypervirulence [22].

The various VFs associated with *P. aeruginosa* are potential targets for vaccine development (Figure 1). Detailed studies of these factors are therefore essential for designing effective strategies to combat infections caused by this pathogen.

## 3. Immunology of *P. aeruginosa* Infections

As an opportunistic pathogen, *P. aeruginosa* triggers a robust immune response while simultaneously developing evasion strategies. The immune response to this pathogen is therefore complex and multifactorial, involving both innate and adaptive immunity.

The innate immune response, which serves as the first line of defense, depends on pattern recognition receptors (PRRs), e.g., Toll-like receptors (TLR), to detect bacterial elements. Upon recognition, TLRs activate a signaling cascade that leads to the release of pro-inflammatory cytokines, tumor necrosis factor (TNF), and interleukins (ILs), and to the recruitment of phagocytic immune cells [35]. *P. aeruginosa* is known to express agonists for TLR2, TLR4, TLR5, and TLR9, which recognize lipopeptides, LPS, flagellin, and unmethylated CpG DNA, respectively [36]. In particular, TLR4 and TLR5, which recognize LPS and flagellin, are essential for triggering the immune response in respiratory infections [37,38]. The phagocytic immune cells activated in this response to respiratory infections include neutrophils, which destroy bacteria through proteolytic enzymes, ROS [39], and macrophages. However, *P. aeruginosa* evades this response by forming biofilms and using T3SS effectors like ExoS, which can lyse macrophages and inhibit the phagocytic absorption of both neutrophils and macrophages [36,40]. Likewise, the ability of this pathogen to form biofilms further enhances its persistence in the host [24,25,41].

**Figure 3:**
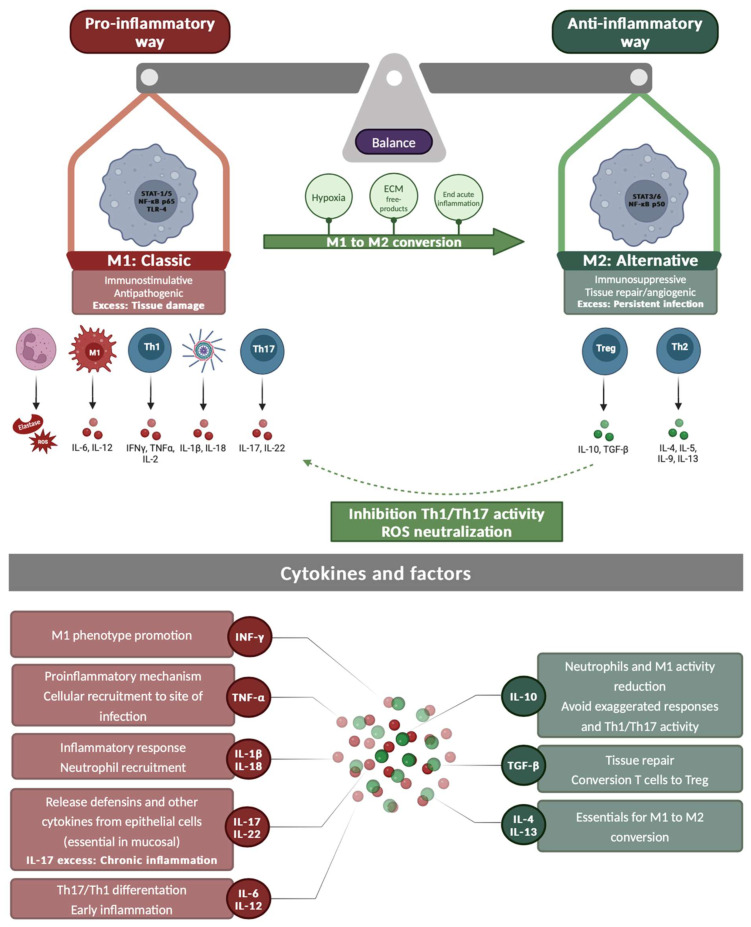
**Upper panel**: Activation pathways of M1 and M2 macrophages. The pro-inflammatory cascade triggered by M1 macrophage activation is shown on the left. The anti-inflammatory cascade resulting from M2 macrophage activation is depicted on the right. The arrows represent connections between pathways. ECM, extracellular matrix; I, inflammasome; IL, interleukin; N, neutrophil; ROS, reactive oxygen species. **Lower panel**: Cytokines and factors released during the inflammatory and anti-inflammatory phases. Pro-inflammatory molecules are shown with a red background and anti-inflammatory molecules are shown with a green background.

On the other hand, the adaptive immune response is activated when antigen-presenting cells (APCs) present *P. aeruginosa* antigens to T lymphocytes, initiating a cascade that activates B lymphocytes to produce specific antibodies. These antibodies facilitate the clearance of bacteria through phagocytosis and/or opsonization [35,39]. However, persistent *P. aeruginosa* cells can survive macrophage attacks and even redirect them toward an anti-inflammatory M2-type response (Figure 3) [42]. Th17 cells, a subtype of CD4+ T lymphocytes, secrete IL-17, IL-21, IL-22, and IL-26, which are crucial cytokines for mucosal immunity, particularly in pulmonary infections [43,44]. IL-17 and IL-22 stimulate the release of pro-inflammatory cytokines from mucosal cells [45] and play a key role in controlling bacterial loads during the early stages of infection. However, Th17 differentiation is controlled by both interferon-gamma (IFN-γ) (Th1) and IL-4 (Th2) [45]. In chronic infections, such as those observed in patients with chronic obstructive pulmonary disease (COPD), excessive release of IL-17 has a dual effect: it contributes to bacterial clearance and can also lead to prolonged inflammation and tissue damage [46]. Other cytokines, such as IL-1β and IL-18, are also responsible for the exacerbated damage in acute pneumonia through activation of inflammasomes [47].

The immune response to *P. aeruginosa* in patients with CF is mediated by exacerbated chronic inflammation and ineffective bacterial clearance. It is characterized by the massive recruitment of neutrophils to the lungs, where elastases and ROS are released. However, these responses cause tissue damage without effectively eliminating the bacteria. The biofilm formation and antigenic variability of *P. aeruginosa* enable the bacteria to evade phagocytosis and antibiotics, thus contributing to the persistence of the infection. The continuous release of cytokines and proteases by neutrophils results in progressive deterioration of tissue, worsening the condition of patients with CF and making *P. aeruginosa* infection a key factor in the progression of lung disease [39,41,48,49]. Further studies are required in regard to achieving elimination of the pathogen from the organism or preventing *P. aeruginosa* infections, for which effective vaccines would play a key role.

## 4. Vaccination Against *P. aeruginosa*

**Figure 4:**
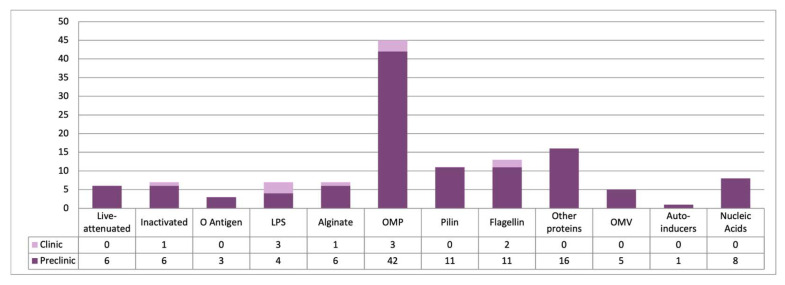
Distribution of vaccine designs by base components. Graphical representation of the number of vaccine designs categorized by their primary base components. LPS, lipopolysaccharide; OMP, outer membrane protein; OMV, outer membrane vesicle.

Historical successes, such as the eradication of smallpox [50] and the development of the SARS-Cov-2 vaccine [51,52] in record time, have inspired both past and future efforts to develop a vaccine against *P. aeruginosa*. Different strategies targeting VFs have been explored for more than 50 years, but no successful vaccine against this pathogen has yet been approved for human use. Figure 4 shows the number of studies conducted using different technologies to develop vaccines against *P. aeruginosa*, including those in the preclinical stages and those that have advanced to the clinical phases. Tables S1–S4 summarize the key characteristics of the experimental vaccines evaluated in different animal infection models, including pneumonia, burns, sepsis, and others. Table S5 lists the vaccine candidates targeting *P. aeruginosa* that have advanced to phase I, II, and III clinical trials.

### 4.1. Live-Attenuated Vaccines

Live-attenuated vaccines use pathogenic strains with reduced virulence while preserving immunogenicity to provoke an effective immune response [53]. Moreover, these vaccine candidates contain multiple antigenic components, which stimulate multiple immune effectors in the host, thereby reducing the selective pressure for the emergence of resistant strains. The development of resistance could occur when vaccine candidates include antigens that are not essential for causing acute pneumonia or system invasion [54]. Unfortunately, this vaccination strategy is highly reactogenic and is associated with side effects such as weight loss and lesions at the injection site in mice [55,56]. While these vaccines often elicit stronger cellular immune responses than inactivated vaccines, providing long-lasting protection, safety concerns, such as the potential reversion to virulence, require careful evaluation and optimization.

#### 4.1.1. Live Auxotrophic Vaccines

Mutants of *P. aeruginosa* PAO1 with a deletion of the *aroA* gene, rendering the strains auxotrophic for aromatic amino acids, have shown some effectiveness in pneumonia models with mice and rabbits [57,58]. Intranasal vaccination (IN) with PAO1 Δ*aroA* induced high titers of opsonizing antibodies and provided protection against homologous LPS strains [57], with no dissemination to other organs observed [58]. Modified PA14 Δ*aroA* strains provided broader protection, inducing IL-17 secreted by T cells [59]. Finally, a multivalent live-attenuated mucosal vaccine with Δ*aroA* strains covering diverse O-antigen epitopes in the O2/O5 serogroup generated multifactorial immunity against *P. aeruginosa* lung infections, with opsonophagocytic antibodies targeting LPS O-antigens, the LPS core, and surface proteins [54].

On the other hand, PAO1 mutants lacking the *murI* gene, which encodes glutamate racemase, generated a D-glutamate auxotrophic strain with attenuated virulence, capable of inducing robust antibody responses and providing protection against heterologous strains [60]. This vaccine elicited IL-17 and IL-4 secretion by T cells, enhancing mucosal immunity when administered IN and providing protection against lethal pneumonia [56]. However, for the PAO1 Δ*murI* mutant, some variants regained D-glutamate auxotrophy. Additionally, D-alanine auxotrophy in the alanine racemase-defective triple mutant PAO1 Δ*murI* Δ*alr* Δ*dadX* improved the safety of the vaccine candidate [55].

A novel live auxotrophic vaccine for p-benzoyl-L-phenylalanine (BzF) demonstrated protection against *P. aeruginosa* sepsis and acute pneumonia, eliciting higher IgG responses than the inactivated PAO1 and showing cross-reactivity against several strains [61].

#### 4.1.2. Recombinant Vaccines with Attenuated Carriers

Recombinant vaccines using attenuated *Salmonella enterica* serovar Thyphimurium (SL3261/pLPS2) as carriers to express *P. aeruginosa* antigens (e.g., LPS O-antigens) have been evaluated in animal models. Oral administration (PO) of the construct expressing the *P. aeruginosa* (PA103) serogroup O11 in acute pneumonia models provided better protection than intraperitoneal (IP) administration and induced strong immunoglobulin G (IgG) and IgA responses [62]. IN administration was also effective, even in patients with corneal lesions and burns [63]. The efficacy of the vaccine was also demonstrated in immunocompromised mice susceptible to pneumonia, improving survival [64]. A similar vaccine expressing the *P. aeruginosa* serotype O9 required a higher dose for immunization but showed comparable efficacy, increasing the IgG2a and IgG2b levels, as well as enhancing the opsonophagocytic killing capacity [65]. However, the same procedure using *Salmonella* Typhimurium SL1344 Δ*wecA*, an attenuated mutant strain with a disruption in the enterobacterial common antigen biosynthesis pathway, failed to protect against IN infection in pneumonia models when administered PO, although it was successful via IP injection [66].

#### 4.1.3. Killed but Metabolically Active (KBMA) Vaccines 

An approach involving the photochemical inactivation of *P. aeruginosa* strains that do not cause disease but retain metabolic activity to elicit a potent immune response has been reported [67]. A KBMA vaccine against *P. aeruginosa* (PA CHA Δ*uvrAB* Δ*exoS* Δ*exoT*) generated high antibody titers and specific T cell activation (Th17, Th1 and Th2). IN administration in lethal pneumonia models yielded a survival rate of 58.3% in immunized mice relative to non-immunized mice [68].

### 4.2. Inactivated Vaccines

Inactivated vaccines utilize dead pathogenic cells, obtained by different techniques, such as inactivation by formaldehyde, heat or hydrogen peroxide treatment. These vaccines are generally safer than live vaccines but tend to generate weaker immune responses, and booster doses are often required [69].

Pseudostat® is a prototype vaccine consisting of *P. aeruginosa* inactivated with paraformaldehyde that was tested in acute respiratory infection models using various administration routes, including PO, intratracheal (IT), intra-Peyer’s patch (IPP) and subcutaneous (SC) routes [70]. The most effective method was IPP, yielding up to 96% bacterial clearance in bronchoalveolar fluid (BAL) and an associated increase in survival rates. High antibody levels were correlated with bacterial clearance, but excessive titers were linked to a poor prognosis in some studies. Remarkably, intestinal mucosal immunization provided some protection against respiratory infections [70]. Pseudostat^®^ reached the phase I clinical trial stage on two occasions, reflecting scientific interest in this candidate. The initial trial demonstrated a reduced bacterial load in sputum and improved lymphocytic immunity [71]. Subsequent studies in healthy volunteers showed the development of specific IgA and IgG responses. Although some adverse effects detected during the study, such as headache, gastrointestinal disorders or upper respiratory tract infections, were not considered clinically significant [72], further development of this vaccine was limited.

Rodgers et al. developed microneedles loaded with heat-inactivated *P. aeruginosa* PAO1, delivering antigens quickly into the skin. This approach reduced the bacterial loads in the lungs and spleen [73]. Additionally, IN administration of a heat-inactivated whole-cell vaccine with Curdlan as an adjuvant reduced the bacterial loads in the nasal lavage and lungs, although it was less effective at resolving lung inflammation than its counterpart targeting *Bordetella pertussis* [74]. The humoral response was shown to play a critical role in this vaccination approach [75].

PAO1 vaccines inactivated by hydrogen peroxide or heat have been evaluated in sepsis models. The surface epitopes were better preserved when hydrogen inactivation was used, generating stronger immunogenic responses. This approach enhanced IgG production and improved IFN-γ and IL-4 secretion, although the cells appeared to release unidentified material, possibly DNA, during vaccination [76].

Bacterial ghosts (BGs), which are empty bacterial envelopes retaining all the surface structures [77], have been tested with *P. aeruginosa* in both oral and topical vaccination. BGs triggered robust humoral and cellular immune responses, with increased phagocytic activity and elevated IFN-γ levels. These vaccines effectively reduced the bacterial loads in the organs and protected against epidermal ulcers in diabetic rat models [78].

Jiang et al. (2023) have recently used X-ray-inactivated *P. aeruginosa* in a murine keratitis model. IN immunization reduced bacterial colonization in the cornea and improved the clinical severity scores following *P. aeruginosa* infection. By contrast, non-immunized mice had increased bacterial loads in the respiratory tract, suggesting translocation of the infection. The vaccine provided cross-protection against heterologous strains, induced M2 macrophage responses to reduce inflammation, and increased anti-inflammatory cytokines such as IL-10 [79].

### 4.3. LPS-Based Vaccines

A heptavalent vaccine, designed in the 1970s, was based on seven immunotypes of antigens capable of inducing cross-protection [80]. Initial safety and antigenicity studies in prison volunteers led to clinical trials under the name Pseudogen^®^, which was used to immunize patients with CF or burns. Despite eliciting increased levels of LPS-specific antibodies, this vaccine caused high levels of toxicity, with no clinical improvement [81]. Phase I and II trials, first with prisoner volunteers and later with burn patients (over 20% total body surface burned), suggested a correlation between antibody titers and infection resistance. However, positive outcomes were not achieved with severe infections [82]. Further trials demonstrated that Pseudogen^®^ prevented sepsis and reduced mortality caused by *P. aeruginosa*, highlighting the critical role of neutrophils and IgG in resistance to infection [83,84]. Optimized dosing further reduced mortality, even in severe burns (over 40% of the body), with an overall reduction of 15.7% and an 86% for *P. aeruginosa*-related sepsis [85]. Despite these findings, inconclusive results in cancer patients and several reported adverse reactions raised safety concerns [86]. In addition, trials in children and adults with acute leukemia and CF also yielded disappointing results due to toxicity concerns [87,88,89].

Around the same time, PEV-01, a polyvalent vaccine combining 16 *P. aeruginosa* serotypes, was developed [90]. In sepsis models, each component induced early protection in mice. Phase II trials showed no toxicity and provision of early protection in healthy volunteers and burn patients [91,92]. However, subsequent studies in CF patients reported the worsening of disease in colonized and vaccinated individuals [93].

Exotoxin A (ETA) is one of the most potent cytotoxins secreted by *P. aeruginosa*. ETA production is regulated by iron and its cytopathic activity stems from inhibiting protein biosynthesis and suppressing pro-inflammatory cytokines, such as TNF-α, IL-10, IL-6, and IL-8, which contribute to the severity of the infection [11]. A combined vaccine (Aerugen^®^) was developed using attenuated ETA and LPS from *P. aeruginosa* 27316 (immunotype 5), adjuvanted with Al(OH)_3_. Experiments in rabbits and mice showed that Aerugen^®^ was non-toxic, elicited anti-LPS and anti-ETA IgG responses, and also protected against fatal sepsis caused by *P. aeruginosa* in a burn-induced sepsis model in mice [94]. Aerugen^®^ advanced to phase III trials. Initial studies in healthy volunteers and non-colonized CF patients demonstrated enhanced immunity to LPS components and robust IgG responses [95,96,97,98]. However, phase III clinical trials with nearly 500 CF patients showed no differences between the vaccinated and control groups, leading to the study being suspended [99].

More recently, a conjugate vaccine composed of LPS from both *Klebsiella pneumoniae* and *P. aeruginosa* was evaluated in a murine pneumonia model, demonstrating 100% survival and highlighting the protective potential of LPS [100].

Immunization with the O-antigen of LPS triggers the production of opsonizing antibodies, which correlate with protection against *P. aeruginosa* infection in animal models and patients during acute infection. However, due to its properties, the O-antigen does not confer broad-spectrum immunogenicity or cross-protection against other serotypes [101,102]. These antigens, which induce a T-independent immune response, are ineffective in protecting young populations or immunocompromised individuals, and they do not generate immunological memory [103]. Due to this diversity of serotypes and the lack of demonstrated clinical efficacy of these vaccine candidates, recent studies have mainly focused on developing vaccines based on conserved epitopes, outer membrane vesicles, or whole cells (inactivated or attenuated).

### 4.4. Alginate-Based Vaccines

The mucoid exopolysaccharide (MEP) or alginate produced by *P. aeruginosa* protects against antibiotics, inhibits the complement system and reduces phagocytosis [104]. These findings suggest that the alginate could be a potential vaccine target. Pier et al. (1990) found that opsonizing antibodies against MEP were effective in models of endobronchial and chronic infections, aiding phagocytosis and reducing the bacterial load in the lungs of immunized mice [105]. However, the vaccine showed limited effectiveness against strains that induce non-opsonizing antibodies. Two alginate-based vaccines from the mucoid *P. aeruginosa* strain 2192, differing in the molecular size of MEP, reached phase I trials but elicited weak opsonizing antibody responses in healthy individuals. Additionally, booster doses failed to enhance immunity, limiting their potential use [106].

To improve the immune response against mucoid strains of *P. aeruginosa*, Cryz and colleagues combined alginate and ETA, demonstrating enhanced opsonizing antibody titers, improved phagocytosis, and partial neutralization of ETA toxicity. While promising for CF patients, this approach carried risks of immune hypercomplex formation, which could aggravate the clinical course of the disease [107]. In animal models, a conjugate vaccine using MEP linked to keyhole limpet hemocyanin (KLH) showed increased IgG titers and cross-reactivity against heterologous mucoid strains. In addition, the vaccine induced opsonizing antibodies in the presence of non-opsonizing antibodies, a common characteristic in CF patients [108]. Although KLH is considered a promising carrier, concerns about allergic reactions and variability have so far limited its widespread use in human vaccine development.

Nanotechnology has also been applied to alginate-based vaccine development. A polylactic-co-glycolic acid (PLGA)-based vaccine conjugated with alginate enhanced opsonic activity and pro-inflammatory cytokine production (TNF-α, IL-4, IL-17A, and INF-γ). In a model of chronic infection, this vaccine significantly reduced the bacterial load in the spleen of immunized mice [109]. At the same time, a combination of alginate with solid lipid nanoparticles (Alg-SLNs), administered via intramuscular (IM) injection, resulted in increased IgG, IgA and IgM titers and the production of opsonophagocytic antibodies. The conjugate provided superior protection against *P. aeruginosa* than alginate alone in an acute pneumonia model [110].

Researchers have recently developed a vaccine using synthetic mannuronic acid alginate glycans conjugated with CRM-197-1, and adjuvanted with Freund’s adjuvant (FA). This vaccine elicited high IgG1 and IgG2b antibody titers and improved survival rates in a murine pneumonia model, reducing the bacterial loads in the lungs and bloodstream for both the mucoid and non-mucoid strains of *P. aeruginosa* [111].

### 4.5. OMP-Based Vaccines

Vaccine candidates based on OMPs typically include antigens with highly conserved and surface-exposed immunogenic domains [9]. Unlike live-attenuated vaccines, OMP-based vaccines are considered safer, as they generate fewer adverse effects, which, when present, tend to be milder [112]. However, they exhibit lower immunogenicity compared to attenuated whole-cell vaccines and are more susceptible to enzymatic degradation and/or mucociliary clearance on mucosal surfaces [9].

OprI, a lipoprotein of the OM of *P. aeruginosa*, has been studied as a vaccine candidate. Finke and colleagues demonstrated that both recombinant OprI expressed in *Escherichia coli* and the purified OprI from *P. aeruginosa* serogroup 12, adjuvanted with Al(OH)_3_, increased survival rates in a sepsis model [113]. Likewise, a chimeric OprI-ExoS protein encapsulated in gelatin nanoparticles enhanced both Th1 and Th2 responses, increasing IgA and IgG titers and cytokines such as IL-4, IL-17, and INF-γ. In a pneumonia model, immunized mice had significantly lower bacterial counts, with slightly better results in those receiving alum-adjuvanted protein than the nanoparticle-based vaccine [114].

OprF, another OMP, has also been extensively studied as a protective immunogen. In a burn model, the OprF protein provided immunity and reduced mortality in infected mice [115]. In a chronic pneumonia model, OprF conjugated with elastase and ETA adjuvanted with Al(OH)_3_ provided protection against *P. aeruginosa* infection, but the combination with elastase A exacerbated lung damage [116]. In acute pneumonia models, recombinant OprF activated dendritic cells (DCs), similarly to native OprF, and demonstrated a superior capacity to reduce bacterial colonization and promote production of IL-10, IL-12p70, INF-γ, leading to a decrease in inflammation [117]. To enhance the immune response to OprF, *P. aeruginosa* epitopes (Epi8) were incorporated into adenoviral (Ad) vectors, and OprF was conjugated with the AdC7-RGD (modified with an arginine–glycine–aspartic acid sequence), promoting robust humoral and cellular responses [118,119,120,121]. The histidine-tagged OprF, adjuvanted with Bacillus Calmette–Guérin (BCG) or alum, increased the IgG1 and IgG2a antibodies and decreased the bacterial counts in the kidneys and lungs. In addition, decreased neutrophil recruitment and reduced tissue damage in the liver of immunized mice were observed [122]. Mayeux et al. generated OprF proteoliposomes, achieving 90% survival in an acute pneumonia model using the CHA strain of *P. aeruginosa*. Moreover, sera containing OprF-induced antibodies provided complete protection as a post-infection treatment [123]. On the other hand, a multi-epitope vaccine derived from OprF conjugated with a TLR agonist and formulated in dipalmitoylphosphatidylcholine (DPPC)/cholesterol (Chol) liposomes, generated a balanced Th1/Th2 response and induced potent opsonophagocytic activity [124].

In chronic pneumonia models, Gomi and colleagues evaluated an adenoviral vector vaccine (AdC7) expressing *P. aeruginosa* OprF fused with an integrin-binding RGD sequence. This vaccine, administered IN, generated strong humoral and cellular immune responses, successfully resolving the infection and decreasing pulmonary bacterial colonization [125].

The combination of OprI and OprF with ETA, adjuvanted with aluminum phosphate, improved survival in a burn model (60% vs. 50% survival with OprF alone) while enhancing phagocytosis and neutralizing ETA’s cytotoxicity [126]. Nanoparticle-based vaccines, such as polyhydroxyalkanoate (PHA) containing a combination of OprI, OprF and AlgE epitopes, administered by the SC route, increased the IgG2c levels and cytokine production (IFN-γ, IL-10, IL-17A, and IL-6), indicating a Th1 immune response [127]. Similarly, a recombinant *Salmonella enterica* vaccine expressing OprF and OprI of *P. aeruginosa* increased the IgG and IgA levels, resulting in a balanced Th1/Th2 response. Following SC administration and subsequent challenge in a pneumoniae model, this vaccine achieved a 77.78% survival rate [128].

Mannose-modified chitosan microspheres have been evaluated to improve the immunity induced by OprF-OprI vaccines. This approach increased the INF-γ and TNF-α levels and also the IgM and IgA titers in mucosal samples following IN administration, providing 75% protection against *P. aeruginosa* infection [129]. The combination of OprF, OprI and flagellin B increased specific titers against all the proteins and enhanced the phagocytosis and reduced bacterial counts in the blood. However, OprF-flagellin B failed to protect against the non-mucoid PAK strain, and its use against mucoid strains was ruled out [130].

The IC43 vaccine, a fusion protein of OprF and OprI (OprF_190–342_–OprI_21–83_), combines the immunogenic properties of both proteins. Jing and colleagues evaluated an IC43 heptamer fused with alpha-hemolysin (HLA) and adjuvanted with alum, achieving 80% survival compared to 30% with IC43 alone, along with a reduced bacterial load and decreased inflammation in the lungs [131]. A systemic formulation of the IC43 candidate was first evaluated in three phase I trials with healthy individuals and burn patients using different doses, demonstrating safety, tolerability, and increased specific IgG levels [132,133,134]. Later, in phase II trials, ICU patients with mechanical ventilation maintained elevated IgG levels over time, although the study lacked statistical power to assess infection and mortality rates [135]. In phase III trials, with a larger sample size, IC43 was well tolerated, but no differences in mortality or survival were observed compared to controls [136]. Similarly, a mucosal formulation of IC43 was tested in phase I trials with healthy individuals, resulting in increased IgG and IgA titers without adverse effects [137]. Phase I/II studies comparing systemic and mucosal formulations showed superior immune responses with the systemic formulation following a booster dose [138,139].

PHA microspheres presenting antigens such as OprF, OprI, AlgE, and PopB (a translocator protein) with alum generated specific immune responses and high survival rates [140]. Biopolymer particles delivering multiple proteins (OprF, OprI, AlgE, OprL, PopB, PilA, PilO, FliC, Hcp1, and CdrA) adjuvanted with Alhydrogel^®^ also conferred protection [141]. The combination of OprF/I with PopB improved the protection against *P. aeruginosa* pneumonia, increasing the IgG titers and survival [142]. Likewise, a combination of OprF-OprI-PopB adjuvanted with granulocyte–macrophage colony-stimulating factor enhanced the production of IFN-γ, IL-17A, and IL-4, leading to 83.3% survival and reduced tissue damage in a burn model in rats [143].

Beyond OprI and OprF, other OM components of *P. aeruginosa* have been explored as vaccine candidates. Wu et al. identified several proteins (PopB, FpvA, FptA, OprL, and PilQ) that induced IL-17 release in vitro. Purified PopB, adjuvanted with Curdlan, demonstrated protective efficacy in an acute pneumonia model caused by *P. aeruginosa*. While PopB generated a strong systemic and mucosal immune response after IN administration, the specific antibodies lacked opsonic killing capacity [144]. Gao et al. also evaluated recombinant OprL with Curdlan, which activated Th1 and Th17 immune responses, reduced inflammation and provided cross-protection against multiple *P. aeruginosa* strains [145]. In another study, OprH produced protective effects against infection when administered in micelles, while other proteins, such as FpvA, HasR, and FoxA, did not [146]. Baker et al. tested a mucosal adjuvant, the double mutant heat-labile toxin of *E. coli* (dmLT), with an OMP vaccine, increasing IgG and IL-17A production and achieving a 53% survival rate in a pneumonia model [147]. In a recent study, an extracellular polysaccharide isolated from *Lactobacillus plantarum* combined with OprH improved cytokine production (IFN-γ, IL-2, and IL-4) and survival rates, although not as effectively as an inactivated vaccine [148].

PcrV, a component of the *P. aeruginosa* T3SS, has been widely evaluated as a vaccine candidate. Golpasha et al. found that a bivalent combination of PcrV and 3-oxo-C12-HSL increased IgG and IgG1 production and led to 86% survival in a burn model, with reduced bacterial colonization in the liver and spleen of immunized mice, although not in the skin [149]. Hybrid proteins like PcrV-ExoS adjuvanted with alum or monophosphoryl lipid A (MPL) improved the immune responses (IgA, IgG and cytokines IFN-γ, IL-4 and IL-17) and reduced the bacterial loads in the bladder and kidneys in a murine bladder infection model with *P. aeruginosa* PAO1 [150]. A recombinant protein composed of PcrV, OprF and OprI produced high IgG titers and 75% survival in a burn mouse model, with significant reductions in bacterial loads in the liver, spleen, kidney, and skin [151]. Furthermore, a multi-epitope PcrV-OmpE vaccine enhanced the antibody responses and the reduced bacterial load in the bladder in a rabbit urinary tract infection model. In silico analysis confirmed its stable interaction with both TLR2 and TLR4 [152].

In acute pneumonia models, Sawa and colleagues demonstrated that PcrV immunization increased IgG titers and reduced mortality, bacterial colonization, inflammation, and lung damage [153]. A trivalent combination of PcrV, OprI and Hcp1 (a protein regulated by T6SS), adjuvanted with alum, provided better protection, higher survival rates, and less tissue damage and inflammation following infection [154]. Further studies evaluating PcrV with different adjuvants (FA, alum, CpG ODN) via the IP route showed that FA provided the highest protection against mortality, while alum and CpG produced more balanced results. When administered IN, CpG improved survival, reduced pulmonary edema and inflammation, and enhanced Ig titers compared to alum in preventing *P. aeruginosa* infection [155]. To improve the PcrV efficacy, Wan et al. identified the dominant domains for the immune response and combined them into a vaccine that increased the IgG and IgA levels, protecting against *P. aeruginosa* lung colonization [156]. Additionally, a recombinant fusion protein (PaF) composed of PcrV, PopB and the A1 subunit of dmLT [157,158,159,160] induced higher IgG and IgA titers, stimulated a Th17 response, and enhanced bacterial elimination from the lungs [158,160]. When comparing different formulations, such as the oil-in-water MedImmune emulsion (ME), chitosan particles, TLR4 agonists and BECC438 (non-toxic lipid A analogue), researchers found BECC438/ME to be the most effective, although it failed to protect against the *P. aeruginosa* serogroup O12, which produces the exolysin A (ExlA) toxin [159]. To provide broader protection, ExlA was include in the vaccine formulation [157].

Th17-dependent epitopes, such as rePcrV and reAmpC, were used to construct a chimeric protein (PVAC) that strongly enhanced IL-17 production and protection against different *P. aeruginosa* strains [161]. IN vaccination with rePcrV adjuvanted with Curdlan improved phagocytosis and protection, as well as immune cell recruitment and IL-17A production [162]. In another study, a PcrV/OprI vaccine encapsulated in ferritin nanoparticles, and administered IM without adjuvant, increased antibody titers and INF-γ-producing lymphocytes, indicating a Th1 profile. This vaccine led to a 60% survival rate and reduced bacterial colonization in the spleen and lungs, and it lowered the pro-inflammatory cytokines (TNF-α, IL-6, IL-12) in a pneumonia model [163]. PomT, a vaccine combining PcrV, OprF and the mutated ETA, demonstrated similar efficacy to PcrV alone in a pneumonia model [164].

In addition to PcrV, the chaperone protein PcrH, which directly binds the PopB translocator, has also been tested [165]. The PopB/PcrH conjugate, adjuvanted with Curdlan and encapsulated in PLGA particles, enhanced Th17 cytokine and significantly reduced bacterial colonization in the lung, improving survival in acute pneumonia models [166]. Another vaccine candidate, LptF (a lipotoxin related to OmpA), elicited a Th2-type response, increasing IgG1 titers and providing protection in acute pneumonia models by reducing the bacterial loads in the lungs and spleen [167]. Furthermore, the chitin-binding protein D (CbpD), associated with T2SS, protected mice in both pneumonia and sepsis models [168].

### 4.6. Pili-Based Vaccines

Pili and related molecules have been explored as vaccine candidates in acute pneumonia models. A recombinant vaccine based on PilA, a type IV pilus protein, adjuvanted with alum and naloxone, stimulated robust humoral and cellular immune responses, increasing IL-4, INF-γ and IL-17 production following SC administration [169]. The survival rates were higher in immunized mice and bacterial counts reduced in the lungs, with elevated IgG and IgG1 levels, suggesting a Th2-biased immune response [169]. A similar vaccine based on the PilY1 epitope (EP 167-193), delivered via a macrophage membrane-coated PLGA formulation (PNPS@M-EP167-193), increased the titers of epitope-specific antibodies, after IM administration, without in vitro toxicity [170].

Pili-based vaccines for *P. aeruginosa* have been less frequently explored in sepsis models. A synthetic peptide consensus vaccine (C1s) targeting the type IV pilus receptor binding domain (RBD) provided cross-protection and improved survival rates, highlighting the importance of preserving the RBD receptor backbone structure in vaccine design [171]. The study also demonstrated that synthetic peptide-based vaccines were more effective than those derived from pilus structural proteins [172].

For burn-related skin infections, Korpi et al. combined flagellin B and PilA, eliciting a robust immune response, with increased IgG1, IL-4, INF-γ and IL-17 production, as well as enhanced opsonophagocytic activity. Immunization improved survival and reduced the bacterial loads in the organs (liver, spleen, bloodstream), although the skin bacterial loads remained unchanged [173,174,175]. Additionally, these antibodies enhanced the phagocytic capacity, inhibited bacterial invasion, and led to 100% survival in passively immunized mice [176]. Another bivalent vaccine, based on FlaB and PilQ (a type IV pilus secretory complex component) and adjuvanted with alum, yielded a survival rate of 83% and reduced the bacterial loads in both the organs and skin [177]. Finally, a trivalent vaccine combining FlaA, FlaB and PilA induced the highest IgG production, strongly stimulated splenocyte proliferation and cytokine production (IL-4, INF-γ, IL-12), and also reduced IL-10 levels. Vaccine administration improved survival and decreased the bacterial loads in the liver and bloodstream but not in the skin [178].

Some pili-based vaccine candidates have been developed without experimental challenges. A liposomal vaccine containing a pilin-derived B-cell epitope and an influenza hemagglutinin-derived T-helper epitope was designed with TLR agonists (Pam3CAG, Pam2CAG) as adjuvants and a mannosylated lipid for DC targeting [179]. The liposomal formulation enhanced the immune response by improving drug targeting and adsorption. In animal studies, the vaccine increased the IgG levels following IP administration and the IgA levels in the pulmonary mucosa after IN administration. The PakSer epitope combined with Pam3CAG elicited a stronger response, while PakCys was more effective with Pam2CAG. However, a delayed immune response and an incomplete immune profile limited the conclusions that could be reached [179]. A chimeric PilQ-PilA vaccine adjuvanted with alum significantly increased the IgG and IgG1 isotype levels, indicating a Th2-biased response. Sera from immunized mice completely inhibited *P. aeruginosa* PAO1 motility [180]. Additionally, the tight adherence pili or Flp combined with the montanide ISA 266 adjuvant, stimulated T cell proliferation, cytokine production (IL-4, INF-γ, and IL-17), and a balanced Th1/Th2 response in murine models [181].

The immunogenicity of PilS2, a type IVb pilin, has recently been evaluated. An in-silico-designed PilS2 chimera, administered with or without alum, enhanced the humoral responses in mice [182]. PilS2 expressed in *E. coli* increased IgG production, with alum enhancing the IgG2b and IgG3 levels [182,183]. The combination with alum also stimulated splenocytes more effectively, increasing the IL-4 levels without altering the IFN-γ and IL-17 levels, and enhanced the phagocytic capacity (99.98% versus 97% without alum) [183]. However, the efficacy of this vaccine remains unproven.

### 4.7. Flagellin-Based Vaccines

In the previous section, several flagellin-based vaccines were discussed [173,175,176,177,178,184]. Early studies from the 1980s demonstrated that flagellar antigens (FAgs) could generate protective antibodies against *P. aeruginosa*, with divalent FAgs providing protection regardless of the strain’s serotype [185,186,187]. The FlaB flagellin, when combined with alginate and IN administered to mice, increased the IgG levels and opsonophagocytic activity [188].

In murine models of acute pneumonia, multivalent vaccines combining FlaA, FlaB and *P. aeruginosa* OMPs (OprF and OprI) induced robust IgG responses, improved bacterial clearance and reduced lung damage, particularly against non-mucoid strains of *P. aeruginosa* [189]. Antibodies against polymeric flagella were more effective, but monomeric flagellin induced superior TLR5 activation, crucial for innate immunity [190]. A vaccine combining FlaA and the polymannuronic acid (PMA) improved the IgG responses, inhibited motility and protected against both non-mucoid and mucoid strains, without impairing TLR5 activation [191]. Bivalent vaccines with flagellins A and B produced significantly higher survival rates in pneumonia models than monovalent vaccines. This combination enhanced the phagocytic capacity and neutrophil recruitment, inhibited motility and promoted a Th2-biased immune response, including elevated IL-17 levels [192]. Furthermore, a chimeric protein combining FlaA, FlaB, PcrV and OprF reduced bacterial colonization in the liver and lungs, with 100% survival rates in mice challenged with both the PAO1 and PAK strains [193].

FlgE, a protein related to the flagellar hook structure, was identified as a promising antigen. When adjuvanted with alum, FlgE elicited a Th2-biased immune response and reduced both the bacterial loads in the organs and inflammation in murine pneumonia models [194]. The conjugated reFliC-FN, a fusion of flagellin type A (FliC) with ferritin using nanotechnology, shifted the immune responses from the Th2 to Th1 profile, increasing survival rates by 50%, reducing bacterial colonization, and providing broader protection against strains with flagellins A and B in acute lung infections [195].

In recent years, O-polysaccharides have gained attention for their immunogenic potential. Choi et al. investigated both native FlaA and its deglycosylated form (dnFlaA). Although both generated similar antibody levels, dnFlaA failed to protect against *P. aeruginosa* in a burn infection model and yielded poor results in motility inhibition assays. These findings highlight the critical role of O-glycans in vaccine efficacy [196].

Two flagellin-based formulations have reached clinical trials. The monovalent vaccine advanced to phase I and II trials in healthy volunteers, and high levels of specific antibodies against *P. aeruginosa* flagellin were reported [197,198]. Subsequently, the bivalent formulation reached phase III trials and was tested in non-colonized CF patients, reducing the colonization rates in the vaccinated patients. Notably, 34% of patients were protected from acute infections and 51% from chronic infections [199].

### 4.8. Other Protein-Based Vaccines

Several less-explored targets for *P. aeruginosa* vaccination include proteins such as elastase, catalase, ETA, and others. Sokol and colleagues investigated elastase epitopes (15 and 42) conjugated to KLH or tetanus toxoid (TT) as carrier proteins [200]. Both formulations generated similar IgG and IgA titers, but no significant reduction in the pulmonary bacterial load was observed in acute pneumonia models in rats, suggesting protection against the proteolytic activity of elastase rather than the bacteria itself [200]. Mutant elastases with reduced proteolytic activity, developed by Kawamoto et al., demonstrated promising protective effects in a sepsis model [201].

Immunization with the catalase KatA protein was evaluated through a single IPP inoculation or IPP followed by an IT booster. The IPP plus IT inoculation elicited a more robust immune response, reducing the bacterial loads in both the BAL and lungs, and increased the activity of phagocytic cells [202]. Later studies also assessed the immunogenic capacity of amidase, aminopeptidase, KatE, and Pa13, although the responses varied depending on the protein used [203]. A peptide vaccine targeting the siderophore FpvA, conjugated with KLH and adjuvanted with Curdlan, promoted the recruitment of DCs and T cells, and induced a Th17-type immune response after IN administration, reducing pulmonary edema and bacterial colonization in a murine pneumonia model [204].

Extensive research on *P. aeruginosa* ETA, a potent toxin that inhibits protein synthesis, has been conducted [205]. A non-toxic ETA variant, engineered by removing Glu-553 from the active site, was administered with FA via SC or IP routes, generating specific antibodies [206]. Another non-toxic ETA variant, ETA Δ576–613, provided protection against *P. aeruginosa* infection in mice when adjuvanted with alum [207]. Additionally, combining this non-toxic ETA variant with pilin enhanced both mucosal and systemic immunity, increasing the IgG and IgA levels in the serum and saliva after IN administration in mice [208]. However, the variability of pilin sequences may affect the potential protective efficacy. ETA combined with flagellin also increased IgG titers and demonstrated high protective efficacy in sepsis models, although its effectiveness against multiple *P. aeruginosa* strains remains untested [209].

The iron-binding periplasmic protein HitA, adjuvanted with BCG or with incomplete Freund’s adjuvant (IFA), showed some potential in reducing the bacterial load and histological damage in the lungs and liver in a sepsis model [210]. Similarly, the HasAp protein adjuvanted with naloxone elicited a Th2-based immune response, reduced the inflammation and bacterial load in the liver, but had limited effects on other organs (kidneys and lungs) [211].

Recent advances in nanotechnology have allowed the development of a PLGA-encapsulated ETA vaccine. This nanovaccine generated significantly higher IgG levels than uncoated ETA and induced a robust Th17 response, reducing bacterial colonization in the spleen in a sepsis model [212]. Similarly, ETA encapsulated in gold nanoparticles and administered with FA produced elevated IgG titers and resulted in a 100% survival rate following a *P. aeruginosa* challenge [213]. A conjugate of detoxified ETA toxoid and LPS with FA, encapsulated in gold particles, increased the IgG levels and survival rates in sepsis models without toxicity [214].

Finally, Cairns and colleagues developed a vaccine system based on the trisaccharide D-rhamnan derived from the A-band of *P. aeruginosa* [215,216]. This glycoconjugate vaccine elicited a good immune response, which was further improved using the diphtheria toxoid CRM197 [217]. The synthetic conjugates of tetra- and pentasaccharides triggered specific immune responses in both rabbits and mice and cross-reacted with LPS from different *P. aeruginosa* serotypes and clinical isolates.

### 4.9. Outer Membrane Vesicle-Based Vaccines

Gram-negative bacteria, including *P. aeruginosa*, release outer membrane vesicles (OMVs) containing proteins, eDNA, RNA, and other molecules [218]. The protein composition of the OMVs varies depending on their biogenesis, and these vesicles can transport bacterial materials to host cells, acting as inflammatory VFs [219]. Similar to live-attenuated vaccines, OMVs contain VFs such as endotoxins (e.g., LPS), which may pose safety-related issues.

OMVs are being explored as vaccine platforms to induce immune responses. Recent studies have evaluated recombinant OMVs (rOMVs) from *Yersinia pseudotuberculosis* carrying *P. aeruginosa* antigens such as PcrV and HitA. When administered via IM injection in murine models, these vaccines generated specific IgG and IgM antibodies, enhanced T cell responses (Th1 and Th17) and increased production of TNF-α, IL-17A and INF-γ [220]. In addition, bacterial colonization in the organs (lung, liver and spleen) was reduced, particularly in the bloodstream [220,221]. In burn infection models, a similar vaccine combining *P. aeruginosa* OMVs with diphtheria toxoid and alum, administered via SC, achieved 100% survival in immunized mice [222]. It also significantly reduced the bacterial loads in the organs (liver, spleen, blood, skin) while decreasing inflammatory cell infiltration by eight-fold. However, three doses of vaccine were required to induce the optimal immune response [222]. With advances in nanotechnology, Noh and colleagues developed cellular nanodiscs derived from *P. aeruginosa* OMVs, which enhanced both cellular and humoral immune responses, protecting against pneumonia by reducing the lung bacterial loads and pro-inflammatory cytokines (IL-12, IL-17A and TNF-α) [223].

Despite these promising results, OMV-based vaccine development is still in the preliminary stages. Recent work by Bi et al. revealed that X-ray irradiation could induce OMV release, which reduced edema, lesions and pulmonary inflammation in a sepsis mouse model. Preventive treatment with irradiated OMVs reduced peritoneal bacterial colonization by 39.5%, while post-infection treatment yielded a 50.9% reduction [224]. A novel approach using hybrid membrane vesicles (HMVs), composed of macrophage membranes fused with bacterial OMVs and incorporated in gold nanoparticles, effectively stimulated B-cell proliferation in the spleen and antibody production. In sepsis and pneumonia models, prophylactic vaccination produced significant reductions in lung bacterial colonization and the levels of pro-inflammatory cytokines (IL-6, TNF-α and IL-1β), resulting in 100% survival over 14 days [225]. Furthermore, Takahara et al. examined the differences in vesicle production between biofilm and planktonic cultures. Biofilm-derived OMVs contained higher concentrations of LPS and phospholipids, suggesting a more pronounced inflammatory profile and more efficient macrophage recognition [226].

### 4.10. Autoinducer Vaccines

Quorum sensing (QS) is a bacterial communication process that relies on extracellular signaling molecules, known as autoinducers. These molecules, which include acyl-homoserine lactones (AHLs), enable bacteria to respond collectively to changes in population density [227] and have been explored as potential vaccine components. In this context, N-3-oxododecanoyl homoserine lactone (3-oxo-C12-HSL) was conjugated to bovine serum albumin (BSA) as a carrier protein to stimulate antibody production in murine models of acute lung infection. This vaccine generated antibody titers over 100-fold higher than those in the controls and improved the survival rates in some vaccinated mice 48 h post-infection after IN challenge. Although administration of the vaccine reduced the TNF-α levels and modulated lung inflammation, it did not effectively decrease bacterial colonization or enhance bacterial clearance by the immune system [228].

### 4.11. Nucleic Acid Vaccines

Nucleic acid vaccines have gained importance in recent years, particularly during the SARS-Cov2 pandemic, due to their safety profile and ability to elicit both humoral and cellular immune responses [229].

OprF has been investigated as a DNA vaccine against *P. aeruginosa* by testing the pVR1020 plasmid in chronic pneumonia models. Immunization reduced the lung lesions and bacterial loads, with strong opsonizing antibodies and a predominant Th2-biased response. Gene gun administration produced higher and faster antibody responses than IM injection [230]. A multivalent DNA vaccine combining OprF, OprI, PcrV and PilA, administered via IM electroporation, reduced the bacterial loads in the lungs and bloodstream in acute pneumonia models, generating a Th1-biased immune response with elevated levels of IFN-γ and microphage inflammatory protein-2 (MIP-2) in BAL, as well as increased phagocytic cell infiltration. In this case, gene gun delivery yielded suboptimal results, highlighting the importance of delivery methods in the efficacy of the vaccine [231]. A bivalent DNA vaccine containing OprF and PcrV epitopes, delivered with a polyaspartamide/polyethylene glycol di-aldehyde (PSIH/PEG DA) hydrogel, allowed controlled antigen release, higher specific IgG titers and cytokine levels (INF-γ, IL-4, TNF-α, IL-2, and IL-17), and improved survival rate (83%) with reduced bacterial loads and inflammation in a pneumonia model [232].

The TLR5 receptor plays a fundamental role in pathogen recognition and activation of innate immune responses by detection of flagellin. However, excessive TLR5 activation can hinder the innate immune response to *P. aeruginosa* [233]. To address this challenge, the researchers engineered a DNA vaccine using a mutant flagellin (FliC R90A) to minimize TLR5 activation. The mutant successfully induced high levels of cross-reactive antibodies against type A (FlaA) and B (FliC) flagellins, while minimizing TLR5 interference. However, the reduction of TLR5 activation may impact long-term immunity [234].

In mouse sepsis models, the recombinant pVAX1-OprF-VP22 vaccine, combining OprF with the VP22 protein from herpes simplex virus type 1, induced higher IgG2a levels (indicating a Th1-biased response) and increased IFN-γ, providing enhanced protection against infection [235]. However, the VP22 protein lost its structural conformation when it fused to the amino terminus of OprF due to the location at this end of its cell-penetrating peptide. In chicken models, divalent and fusion vaccines combining *oprL* and *oprF* genes of *P. aeruginosa* provided 80% protection and induced high IFN-γ and IL-2 levels, while monovalent vaccines were less effective (40–50% protection) [236]. Further optimization of the OprL/OprF divalent vaccine dosage revealed that a dose of 100 µg was optimal [237]. A trivalent vaccine combining OprL, OprF and FlgE improved the survival rates by 10%, reaching 90% protection after challenge in chickens. However, its efficacy was slightly lower compared to an inactivated vaccine control [238].

Two mRNA vaccines encoding PcrV and a fusion protein OprF-I have recently been evaluated in murine models of burns and sepsis [239]. The PcrV-based vaccine generated a higher specific IgG titer than the OprF-I vaccine, although the combined vaccine enhanced both responses. The mRNA-PcrV vaccine was more effective in activating CD4+ T cells, promoting IL2 and IL4 secretion, opsonizing antibody production and also activating IFN-γ secreting cells. However, neither vaccine significantly activated CD8+ T cells. While all the vaccines provided protection against different *P. aeruginosa* strains by reducing bacterial colonization in the organs and inflammation, the mRNA-PcrV and the combined vaccine were the most effective against severe *P. aeruginosa* infections [239].

## 5. Conclusions and Future Prospects

*P. aeruginosa* is an important pathogen that poses a serious health threat, especially to individuals with CF and bronchiectasis, due to its ability to infect the respiratory tract. Additionally, *P. aeruginosa* is one of the most prevalent pathogens in ICU infections associated with mechanical ventilation and nosocomial infections. It has a broad clinical spectrum, affecting both immunocompetent patients and especially those with some degree of immunosuppression. Furthermore, it easily develops resistance to antimicrobials, including next-generation drugs, and exhibits notable virulence. This pathogen has attracted great attention from the scientific community over the past five decades, with efforts aimed at developing effective antimicrobials and vaccines. Regarding vaccines, a broad spectrum of vaccine strategies has been developed; however, due to safety concerns and limited evidence of efficacy against chronic pulmonary infections, few vaccine candidates have progressed to clinical trials. To date, no vaccine against *P. aeruginosa* has received marketing authorization approval. Despite this, previous studies have provided valuable insights into the mechanisms that can guide the future development of vaccines. Understanding how *P. aeruginosa* interacts with the immune system is crucial for designing vaccines that elicit both humoral and cellular immune responses.

Most vaccine candidates reviewed have been effective in increasing the serum IgG antibody levels specific to *P. aeruginosa*, regardless of the administration route. However, for optimal protection, especially against pathogens targeting mucosal surfaces, vaccines should prioritize stimulating IgA production at these sites. The Th17 immune responses are particularly promising, as they enhance mucosal immunity through cytokine production and neutrophil recruitment, while the Th1 responses triggered by specific adjuvants, such as alum, combined with naloxone or adenovirus vectors, may offer protection against acute infections.

Whole-cell vaccines, including inactivated or live-attenuated variants, and particularly auxotrophic variants, have shown high immunogenicity and induction of the cellular immune response, making them effective against *P. aeruginosa*. However, challenges such as toxicity from the bacterial LPS remain. Protein-based vaccines, especially those targeting OMPs, represent a promising alternative due to their conservation, ease of production, and surface accessibility. The use of live-attenuated bacterial vectors such as *Salmonella* to boost mucosal immunity is also being explored. However, chronic infections, especially in CF patients, require further study to assess the vaccine effectiveness. To improve vaccine safety, several strategies are being considered, including optimized antigen delivery and novel adjuvants like dmLT, which promote Th17-type responses. Mucoadhesive polymers could enhance antigen retention at mucosal sites, improving the efficacy, reducing the required antigen dose and minimizing the associated side effects. Nanoparticle-based platforms hold great promise for enhancing mucosal immunity by targeting respiratory tissues. These platforms can improve antigen stability, increase presentation to immune cells, and enable the co-delivery of multiple antigens for broader protection. DNA vaccines also offer flexibility in encoding multiple targets and could complement other vaccine strategies. RNA-based vaccines, which proved highly effective during the COVID-19 pandemic, present a novel approach for bacterial infections like *P. aeruginosa*. These vaccines can be rapidly designed to include multiple antigens, providing greater safety and higher efficacy and versatility than traditional vaccine methods. mRNA vaccines could address challenges such as antigen variability and downregulation in chronic infections.

Developing a vaccine against *P. aeruginosa* presents a significant challenge for researchers due to the wide range of VFs produced by the bacterium, as well as its remarkable adaptability and resistance mechanisms. However, the growing threat of multidrug-resistant strains and the vulnerability of high-risk populations highlight the urgent need for a solution. Producing a successful vaccine against *P. aeruginosa* may be possible in the near future by the use of multivalent strategies, including advanced technologies such as nanotechnology and RNA-based platforms, and by obtaining a more detailed understanding of host–pathogen interactions.

## Data Availability

No new data were created in this study.

## Supplementary Materials

The following supporting information can be downloaded at https://www.mdpi.com/article/10.3390/ijms26052012/s1.
