# Supplementary material for: Pseudomonas aeruginosa Vaccine Development: Lessons, Challenges, and Future Innovations
Source: Int J Mol Sci. 2025 Feb 25;26(5):2012. doi: 10.3390/ijms26052012 (PMC11900337; doi:10.3390/ijms26052012)
Supplement: Supplementary file 1 [file ijms-26-02012-s001.zip › ijms-3447921-supplementary.pdf]

Material supplementary

Review

Pseudomonas aeruginosa Vaccine Development: Lessons, Challenges, and Future Innovations

Rebeca Santamarina-Fernández [1], Víctor Fuentes-Valverde [1,2,3], Alis Silva-Rodríguez [1], Patricia García [1,2], Miriam Moscoso [1,2]* and Germán Bou [1,2,4]

[1] Servicio de Microbiología, Instituto de Investigación Biomédica de A Coruña (INIBIC), Complexo Hospitalario Universitario de A Coruña (CHUAC), Sergas. 15006 A Coruña, Spain
[2] CIBER de Enfermedades Infecciosas (CIBERINFEC), Instituto de Salud Carlos III, 28029 Madrid, Spain
[3] Área de Medicamentos Biológicos, Agencia Española de Medicamentos y Productos Sanitarios (AEMPS), 28022 – Madrid, Spain
[4] Departamento de Fisioterapia, Medicina y Ciencias Biomédicas, Universidad de A Coruña, 15006 A Coruña, Spain
* Author to whom correspondence should be addressed. Mirian.Moscoso.Naya@sergas.es

Note: All references listed in the Supplementary Tables are also included in the reference list of the main text.

Table S1. Preclinical vaccine candidates for *P. aeruginosa* assessed in murine pneumonia models

Vaccine types		Antigen and adjuvant	Administration route	Experimental model	Outcomes	Year(s)	Reference
Whole cells	Live-attenuated	PAO1 Δ*aroA*, serogroup O2/O5	IN or IP	Acute pneumonia	Th1-type immune response profile	2002-2011	[54,57,58]
		PA14 Δ*aroA*	IN	Acute pneumonia	Th17-mediated immune response	2008	[59]
		PAO1 Δ*murI*	IN, SC, IM	Acute pneumonia	Increased serum IgG, IgA and sIgA in mucosa. Th17-type immune response	2017-2020	[56,60]
		PAO1 Δ*murI* Δ*alr* Δ*dadX*	IN	Acute pneumonia	Increased serum IgG, IgA and sIgA in mucosa. Improved safety	2022	[55]
		BzF amino acid	IN/IP	Acute pneumonia/Sepsis	Elevated IgG levels and protection against lethality, cross-reactivity	2024	[61]
		PA CHA Δ*uvrAB* Δ*exoS* Δ*exoT*	SC	Acute pneumonia	Induced a mixed Th1/Th17 immune response	2018	[68]
Subunits	Inactivated	PA inactivated with PFA (Pseudostat®)	PO, IT, SC, IPP	Acute pneumonia	IPP most effective in protecting against infection	1994	[70]
		Heat-inactivated PAO1 in microneedles	ID	Acute pneumonia	Reduced bacterial load in the lungs and spleen	2018	[73]
		Heat-inactivated PAO1 co-administered with Curdlan	IN	Acute pneumonia	Th17-type immune response	2020	[74]
	O Antigen	PA O-antigen in *Salmonella* Typhimurium, attenuated	PO, IP, IN	Acute pneumonia/Corneal infection/Burn	PO enhances IgG and IgA responses. Effective in leukopenic and neutropenic mice	2004-2008	[62–64]
		PA O9 antigen in *Salmonella*	IN	Acute pneumonia	Increased levels of IgG2a and IgG2b	2023	[65]
		Salmonella enterica Δ*wecA* expressing PA serotype O-antigen O11	IP, PO	Acute pneumonia	PO was not effective. IP generated specific antibodies and significant opsonizing activity	2016	[66]
	LPS	LPS from PA and *K. pneumoniae* with alum adjuvant	IP	Acute pneumonia	Increased survival rate	2022	[100]

**Subunits**	**Alginate**	Mucoid exopolysaccharide	IM	Chronic pneumonia and endobronchial infection	Increase in antibody titers and reduced lung bacterial load. Not effective against opsonizing strains	1990	[105]
		Alginate conjugated with PLGA nanoparticles	IM	Chronic pneumonia	Produced proinflammatory cytokines (TNF-α, IL-4, IL-17A, and INF-γ)	2021	[109]
		Alg-SLN	IM	Acute pneumonia	Improved immune response compared to the use of Alg alone (increased IgG, IgA and IgM)	2021	[110]
		Synthetic tetrasaccharide of mannuronic acid + CMR-197-1 adjuvated with FA	SC	Acute pneumonia	Increased IgG1 and IgG2b levels and improved survival rate	2024	[111]
	**OMP**	OprI with ExoS encapsulated in gelatin nanoparticles with $Al(OH)_3$ adjuvant	IN	Acute pneumonia	Th1 and Th2 responses	2024	[114]
		OprF, elastase, ETA, as monotherapy or in combination. Adjuvant: $Al(OH)_3$	IM	Chronic pneumonia	Increased IgG titers	1993	[116]
		OprF-stimulated DCs	IP	Acute pneumonia	Increased IL-10, IL-12p70 and INF-γ, decreased IL-4	2010	[117]
		OprF expressed in adenovirus (Ad.OprF.Epi8)	SC	Acute pneumonia	Increased serum IgG	2005	[118]
		OprF derived via adenovirus (Ad5)	IM or IT	Acute pneumonia	Increased IL-17 and IL-4	2011	[119]
		OprF expressed in adenovirus with RGD modification (Ad.OprF.RGD.Epi8)	SC or IM	Non-lethal pneumonia	Improved protective capacity	2013	[120]
		OprF transported in Ad5 with RGD modification	IT, IPL, IM	Acute pneumonia	Improved protective capacity	2007	[121]
		N-terminal domain of OprF, with BCG and $Al(OH)_3$	SC	Acute pneumonia	Increase IgG1 and IgG2a and decrease in liver damage	2020	[122]
		OprF proteolisosomes	SC	Acute pneumonia	Increased antibody levels pre- and post-infection and efficacy	2021	[123]
		Fusion protein $OprF_{190-342}$ – $OprI_{21-83}$ expressed in attenuated *S. enterica*	SC or PO	Acute pneumonia	SC immunization with a greater increase in IgG and IgA in intestine and lungs than PO	2015	[128]
		OprF-OprI in chitosan microspheres	IN	Acute pneumonia	Th1 response	2015	[129]

**Subunits**	**OMP**	OprF and OprI, in combination or with flagellin B	SC	Acute pneumonia	Specific antibody titers and improved phagocytic capacity	2018	[130]
		IC43 heptamer (OprF$_{190-342}$ - OprI$_{21-83}$), with adjuvant $Al(OH)_3$	IM	Acute pneumonia	IgG1 titers higher than IgG2, Th2 type response	2020	[131]
		OprF/OprI/AlgE and PopB in PHA particles, adjuvanted with $Al(OH)_3$	SC	Acute pneumonia	Th2 response	2021	[140]
		Biopolymer particles coated with OprF/I-AlgE, or PopB antigen, or epitopes of OprF, OprI, AlgE, OprL, PopB, PilA, PilO, FliC, Hcp1, and CdrA. With or without gel adjuvant (Alhydrogel®)	IN, IM	Acute pneumonia	Specific immune response. Combination showed better results	2022	[141]
		PopB in combination with OprF/I	IN or SC	Acute pneumonia	Th17 response	2022	[142]
		PopB, FpvA, FptA, OprL, and PilQ proteins co-administered with Curdlan adjuvant	IN	Acute pneumonia	Ac-independent and Th17 response-dependent protection	2012	[144]
		OprL$_{22-168}$, in combination with Curdlan	IN	Acute pneumonia	Th17 response	2017	[145]
		OprH,, transported in micelles	IN	Acute pneumonia	Protective capacity	2018	[146]
		OMP, co-administered with dmLT	ID	Acute pneumonia	Anti-PA IgG in lungs of vaccinated mice, but not IgA. Th17-type response	2019	[147]
		PcrV	IN	Acute pneumonia	Increased IgG titers and decreased tissue damage/inflammation	1999	[153]
		Trivalent vaccine (PcrV$_{28-294}$- OprI$_{25-83}$-Hcp1$_{1-162}$, with $Al(OH)_3$	IM	Acute pneumonia/Burn	Increased IFN-γ and IL-17A production by splenocytes in vaccinated mice. High serum IgG titers	2017	[154]
		PcrV, adjuvanted with CpG ODN or $Al(OH)_3$	IN, IP	Acute pneumonia	Increased IgG and IgA titers in BAL, higher in mice immunized with PcrV-CpG. Th1-type response	2018	[155]
		PcrV$_{NH}$ domains N-ter (Met1-Lys127) and H12 (Leu251-Ile294) of PcrV, adjuvated with $Al(OH)_3$.	IM	Acute pneumonia	Th1 and Th17 responses	2019	[156]

Subunits	OMP	Chimera of PcrV, PopB and dmLT subunit A1. Formulated in chitosan particles and ME/BECC438	IN	Acute pneumonia	Stimulated IL17A secretion	2020-2024	[157–160]
		rePcrV, administered with Curdlan adjuvant	IM, IN	Acute pneumonia	Th17 response	2021-2022	[161,162]
		PcrV-OprI encapsulated in ferritin nanoparticles	IM or IN	Acute pneumonia	Th1 immune profile	2023	[163]
		PomT (PcrV-OprF-mETA)	SC	Acute pneumonia	Survival rates comparable to PcrV use	2023	[164]
		PopB and PcrH encapsulated in PLGA nanoparticles	IN	Acute pneumonia	Th17 type response. Increased CD4+ IL-17+ T cells in lungs and splenocytes	2018	[166]
		LpTF	IM	Acute pneumonia	IgG1-Th2 response	2023	[167]
		CbpD	IP	Acute pneumonia/Sepsis	Higher IgG response and improved protective capacity	2023	[168]
	Pilin	PilA with $Al(OH)_3$	SC	Acute pneumonia	Protection associated with an increase in IL-17 and INF-γ	2016	[169]
		PilY1 epitope ($Ep_{167-193}$) in PLGA particle coated with macrophage membrane	IM	Acute pneumonia	Th2 response	2022	[170]
	Flagellin	Multivalent flagellin A, B and OprF-OprI	IM	Acute pneumonia	Elevated IgG1/IgG2a ratio, Th2-type response	2009	[189]
		Flagellum and flagellin	IN	Acute pneumonia	Increase in opsonizing antibody titers	2010	[190]
		Flagellin A conjugated to poly-mannuronic acid	IN	Acute pneumonia	Increase in antibody titers	2011	[191]
		Flagelina A y B	IN	Acute pneumonia	Protection dependent on Th17 response	2017	[192]
		Flag B + FlagA + PcrV+ OprF	IM	Acute pneumonia	Phagocytic antibodies generation and increased survival rate	2023	[193]
		FlgE, adjuvated with $Al(OH)_3$.	IM	Acute pneumonia	Th2 dependent response	2021	[194]
		reFliC-FN (Ferritin nanoparticles)	IM	Acute pneumonia	Th1 response	2023	[195]
		Elastase, LasB, conjugated with KLH or TT, combined with FA	IM + SC	Acute pneumonia	Increased IgG and IgA levels in serum and BAL	2000	[200]
		KatA, in combination with FA	IPP + IT	Acute pneumonia	Increased IgG and IgA levels in serum and BAL	2000	[202]

Subunits	Other protein	Azurin, ACP, amidase, aminopeptidase, catalases KatE and KatA	IPP + IT	Acute pneumonia	Increased IgG and IgA levels in serum and BAL	2009	[203]
		Extracellular region of the siderophore FpvA conjugated to KLH. Curdlan as an adjuvant	IN	Acute pneumonia	Th17 response	2019	[204]
		AdC7OprF.RCG	IN	Chronic pneumonia. Post-exposure immunization therapeutic vaccine strategy	Increased IL-17A expression in lungs of vaccinated mice	2017	[125]
	OMV	Attenuated PA with modified LPS overexpressing the PcrV-HitA fusion protein	IM	Acute pneumonia	Humoral and Th17 type response	2021	[220,221]
		Cellular nanodiscs derived from OM of PA	SC	Acute pneumonia	Reduced levels of IL-6, IL-12, IL-17A and TNF-α	2022	[223]
		HMV (Macrophage membrane vesicles and bacterial OMs)	SC	Pneumonia/Sepsis	Less lung damage and colonization/ B cell proliferation and increased antibody titers	2024	[225]
	Auto-inducers	3-oxo-C_{12}-HSL-BSA, administrated with FA	SC	Acute pneumonia	Increase in serum anti-3-oxo-C12-HSL-BSA IgG	2006	[228]
Nucleic acid vaccines	DNA	*oprF* gene cloned into plasmid pVR1020	BG-ID	Chronic pneumonia	Increased serum IgG levels with IgG1 predominance	2001	[230]
		DNA of *oprF-oprI*, *pcrV*, *pilA* genes	IM-EP	Acute pneumonia	Th1 type response. High IgG, IgG1, IgG2a titers	2006	[231]
		Bivalent DNA vaccine OprF-PcrV with PSIH/PEGDA hydrogel	IT	Acute pneumonia	Mixed Th1/Th2/Th17 immune response	2024	[232]
		DNA encoding FliC R90A or FlaA	IM-EP	Acute pneumonia	Increased serum IgG levels	2007	[234]

3-oxo-C_{12}-HSL-BSA, N-3-oxododecanoyl homoserine lactone with bovine serum albumin; ACP, acyl carrier protein; BAL, bronchoalveolar fluid; BCG, Bacillus Calmette-Guérin; BG-ID, plasmid vaccine delivered by biolistic (gene gun) ID inoculation; BzF, p-benzoyl-L-phenylalanine; DCs, dendritic cells; dmLT, double mutant heat-labile toxin of *E. coli*; ETA, exotoxin A; FA, Freund's adjuvant; ID, intradermic route; Ig, immunoglobulin; IM, intramuscular route; IM-EP, IM electroporation method; IN, intranasal route; IP, intraperitoneal route; IPL, intrapleural route; IPP, intra-Peyer's patch route; IT, intratracheal route; KLH, keyhole limpet hemocyanin; LPS, lipopolysaccharide; LpTF, lipotoxin F; ODN, oligodeoxynucleotide; OMP, outer membrane protein; OMV, outer membrane vesicle; PA, *P. aeruginosa*; PFA, paraformaldehyde; PHA, polyhydroxyalkanoate; PLGA, polylactic-co-glycolic acid; PO, oral route; PSIH/PEG DA, polyaspartamide/polyethylene glycol di-aldehyde; RGD, modification incorporating the arginine-glycine-aspartic acid sequence; SC, subcutaneous route; sIgA, secretory immunoglobulin A; SLN, solid lipid nanoparticles; TT, tetanus toxoid.

Table S2. Preclinical vaccine candidates for *P. aeruginosa* assessed in burn infection models

Vaccine types		Antigen and adjuvant	Administration route	Experimental model	Outcomes	Year(s)	Reference
Subunits	O Antigen	PA O-antigen in *Salmonella* Typhimurium, attenuated	PO, IP/IN	Burn/Corneal infection/Acute pneumonia	IN is effective in burn/corneal infections	2004-2007	[62,63]
	LPS	LPS from seven serotypes of PA (Pseudogen®)	IM	Burn	Higher antibody titers	1969-1971	[80,81]
		LPS isolated from PA 27316 conjugated with ETA and adjuvanted with $Al(OH)_3$	IM	Burn	Increased anti-LPS IgG and protective activity against fatal sepsis	1986	[94]
	OMP	OprF	IM	Burn	Decreased mortality and production of reactive antisera	1990	[115]
		OprF, OprI, ETA fusion protein, adjuvanted with $AlPO_4$	IP	Burn	Opsonizing antibodies	1999	[126]
		OprF-OprI-PopB adjuvated with GMCSF	SC	Burn	Increased IL-17A and IL-4 levels	2022	[143]
		PcrV conjugated with 3oxo-C_{12}-HSL, combined with FA	SC	Burn	Increase in serum antibody titers	2015	[149]
		Chimera $OprF_{185-350}$ –$OprI_{22-83}$ – PcrV	SC	Burn	Increase in IgG titers	2020	[151]
		Trivalent vaccine ($PcrV_{28-294}$- $OprI_{25-83}$-Hcp_{11-162}, with $Al(OH)_3$	IM	Burn/Acute pneumonia	Increased IFN-γ and IL-17A by splenocytes in vaccinated mice. High serum IgG titers	2017	[154]
	Pilin	PilA and flagellin B	SC	Burn	Protection associated with IL-17, INF-γ and IL-4	2016-2017	[173,176]
		PilA alone or with alum	SC	Burn	Th2 response, increased IgG1, IL-4 and INF-γ	2015	[174]
		PilQ and flagellin type B	SC	Burn	Increased IL-17 and IL-4 in stimulated splenocytes	2018	[177]

Subunits		Flag A + FlagB + PilA	SC	Burn	Potentiation of IL-12/IL-10 and specific antibodies	2020	[178]
	Flagellin	Flag A and FlagB	SC	Burn	Specific antibodies and increased IL-4 and IL-12, low IL-10	2016-2017	[175,184]
		Bivalent flagella vaccine	IM	Burn	Production of antibody binders	1982-1986	[185–187]
		Deglycosylated FlaA vs native FlaA	IM	Burn	Poor immune response, highlighting the importance of glycosylation	2024	[196]
	OMV	OME with diphtheria toxin and adjuvanted with $Al(OH)_3$	SC	Burn	Less infiltration of inflammatory cells	2022	[222]
Nucleic acid vaccines	mRNA	mRNA-OprF-I and mRNA-PcrV	IM	Burn/sepsis	Increased serum IgG titers	2023	[239]

ETA, exotoxin A; FA, Freund's adjuvant; GMCSF, granulocyte-macrophage colony-stimulating factor; Ig, immunoglobulin; IM, intramuscular route; IN, intranasal route; IP, intraperitoneal route; LPS, lipopolysaccharide; OMP, outer membrane protein; OMV, outer membrane vesicle; PA, *P. aeruginosa*; PO, oral route; SC, subcutaneous route.

Table S3. Preclinical vaccine candidates for *P. aeruginosa* assessed in murine sepsis models

Vaccine types		Antigen and adjuvant	Administration route	Experimental model	Outcomes	Year(s)	Reference
Whole cells	Attenuated	BzF amino acid	IP/IN	Sepsis/Acute pneumonia	Increased IgG levels and protection against lethality, cross-reactivity	2024	[61]
Subunits	Inactivated	PAO1 inactivated by H_2O_2	SC	Sepsis	Th2 and Th1 responses	2019	[76]
	LPS	LPS isolated from 16 strains (PEV-01)	IP	Sepsis	Protective response for each vaccine separately	1997	[90]
	OMP	OprI, formulated with $Al(OH)_3$	IP	Sepsis	OprI purified and expressed present protective capacity	1990	[113]
		OprH with *L. plantarum*	SC	Sepsis	Increased INF-γ IL-2 IL-4 levels	2023	[148]
		CbpD	IP	Sepsis/Acute pneumonia	Higher IgG response and protective capacity	2023	[168]
	Pilin	C1s (synthetic peptide vaccine) targeting the receptor of type IV pili	IP	Sepsis	Synthetic peptides improved survival and induced cross-reactivity	2007-2009	[171,172]
		Recombinant elastase (LasB)	SC	Sepsis	Protective effects and importance amino acids determined	1993	[201]

Subunits	Other proteins	ETA and flagellin fusion protein	SC	Sepsis	Opsonizing antibodies	2015	[209]
		HitA, iron-binding periplasmic protein, BCG adjuvanted	SC, IM	Sepsis	Increased IgG2a titers, Th1 response	2019	[210]
		HasAp with naloxone adjuvant	SC	Sepsis	Th2 response and lower IgG titers	2022	[211]
		ETA encapsulated in PLGA nanoparticles	IM	Sepsis	Elevated serum IgG titers. Th17-type response	2019	[212]
		ETA with gold nanoparticles	SC	Sepsis	Increased IgG titers and improved survival rates	2021	[213]
		Detoxified LPS combined with ETA. Formulated with gold nanoparticles, with FA	SC	Sepsis	Safe, increased IgG titers	2021	[214]
	OMV	X-ray irradiated OMV	IP	Sepsis	Pre- and post-infection treatment	2024	[224]
		HMV	SC	Sepsis/Pneumonia	B cell proliferation and increased antibody titers/ Less tissue damage and colonization in lungs	2024	[225]
Nucleic acid vaccines	DNA	Recombinant DNA encoding OprF and HSV-1 VP22 protein	IM	Sepsis	Th1 response	2016	[235]
		DNA encoding OprL and OprF, applied as monovalent or bivalent immunization	IM	Sepsis	Increased serum IgG, especially in those immunized with bivalent candidate. Increased IFN-γ and IL-2 levels	2018-2021	[236,237]
		DNA encoding OprI, OprF and FlgE applied as trivalent formulation	IM	Sepsis	Increased INF-γ, IL-2, and IL-4 levels	2022	[238]
	mRNA	mRNA-OprF-I and mRNA-PcrV	IM	Sepsis/burn	Increased serum IgG titers	2023	[239]

BCG, bacillus Calmette-Guérin; BzF, p-benzoyl-L-phenylalanine; ETA, exotoxin A; FA, Freund's adjuvant; HMV, hybrid membrane vesicles composed of macrophage membranes fused with bacterial OMVs and incorporated in gold nanoparticles; Ig, immunoglobulin; IM, intramuscular route; IN, intranasal route; IP, intraperitoneal route; LPS, lipopolysaccharide; OMP, outer membrane protein; OMV, outer membrane vesicle; SC, subcutaneous route; PA, *P. aeruginosa*; PLGA, polylactic-co-glycolic acid.

Table S4. Preclinical vaccine candidates for *P. aeruginosa* assessed in other animal models

Vaccine types		Antigen and adjuvant	Administration route	Experimental model	Outcomes	Year(s)	Reference
Whole cells	Inactivated	Ghost PA	PO	Oral and topical challenge	Elevated INF-γ and increased cellular/humoral response	2022	[78]
		X-ray inactivated PA	IN	Keratitis	Reduced corneal bacterial colonization	2023	[79]
Subunits	O Antigen	PA O antigen in *S.* Typhimurium, attenuated	IN	Corneal infection/Acute pneumonia/Burn	Increased IL-10 and antibody response. Macrophages M2 activation	2004-2007	[62,63]
	Alginate	Mucoid exopolysaccharide, alginate, conjugated with ETA, adjuvanted with $Al(OH)_3$	IM	No challenge	Increased anti-alginate and anti-ETA antibody titers	1991	[107]
		Mucoid exopolysaccharide conjugated with KLH	SC	No challenge	Increased IgG titers with opsonizing capacity	2003	[108]
	OMP	Multiepitope OprF derived with toll-receptor agonist in DPPC/Chol liposomes	IP	No challenge	Balanced Th1/Th2 profile and opsonizing response	2023	[124]
		OprF, OprI, AlgE in PHA nanoparticles, with adjuvant $Al(OH)_3$	SC	No challenge	Th1-type response. Elevated of IFN-γ and IgG2c levels	2017	[127]
		Chimera of PcrV and the C-terminus of ExoS with $Al(OH)_3$ or MPL adjuvant	SC	Bladder infection	Th17-type response and increased IgA and IgG titers in mucosa	2022	[150]
		PcrV and OmpE multiepitope vaccine with FA	SC	Transurethral infection	Higher antibody response	2024	[152]
	Pilin	Pili protein epitope B formulated in liposome and adjuvanted with Pam3CAG, TLR4 agonist	IP e IN	No challenge	IP immunization did not produce IgA titers in the lung, but IN did.	2009	[179]
		PilQ/PilA chimera adjuvanted with $Al(OH)_3$	SC	No challenge	Th2-type response	2017	[180]

Subunits		Low molecular weight pili (Flp), in combination with Montanide ISA 266, an oily adjuvant	IP	No challenge	Increase in INF-γ and IL-17. Elevated IgG titers	2020	[181]
		PilS2 with or w/o alum	SC	No challenge	With Alum enhanced IgG2b and IgG3 responses	2023-2024	[182,183]
	Flagellin	Alginate-conjugated flagellin type B	IN	No challenge	Increase in antibody titers	2017	[188]
	Other proteins	ETA E553Δ, combined with FA	SC, IP	No challenge	Increased serum antibody titer	1988	[206]
		ETA A (Δ576-613), adjuvanted with $AlPO_4$	SC	Challenge by SC injection of ETA	Increase in anti-ETA antibody titers	1999	[207]
		Chimera of ETA and PilA	IN	No challenge	Increase in IgG, IgA and sIgA titers	2005	[208]
		D-rhamnan derived from A-band PA	IP/SC	No challenge	Specific immune response and recognition of clinical isolates	2022-2024	[215–217]

DPPC/Chol, dipalmitoylphosphatidylcholine/cholesterol; ETA, exotoxin A; FA, Freund's adjuvant; Ig, immunoglobulin; IM, intramuscular route; IN, intranasal route; IP, intraperitoneal route; KLH, keyhole limpet hemocyanin; MPL, monophosphoryl lipid A; OMP, outer membrane protein; w/o, without; PA, *P. aeruginosa*; Pam3CAG, triacetylated Pam3cysteinyl-alanyl-glycine; PO, oral route; SC, subcutaneous route; sIgA, secretory immunoglobulin A.

Table S5. Vaccines against *P. aeruginosa* infections in clinic trials

Classification	Antigen	Phase	Immunization				Study population	Results	Year(s)	Study
			No. of doses	*Admin. route*	*Dosage*	*Adjuvant*				
**Inactivated cell**	Pseudostat®	I	3	PO	10^{11} killed PA	w/o	9 bronchiectasis pt.	Fewer bacterial counts, enhanced of lymphocytic immunity	1997	[71]
		I	2	PO	150 mg	w/o	30 healthy indiv.	Opsonizing IgG and IgAs, 20 adverse events	2006	[72]
**LPS**	Pseudogen®, LPS from 7 PA serotypes	I*	3	IM	4.3-11.1 µg/kg	w/o	40 burn pt.	Not effective in cases of severe infections	1969	[82]
		II*	Various regimes	IM/SC/ ID+SC/ ID+IM	10-280 mcg/kg	w/o	72 burn pt.	Prevention of sepsis and reduction in mortality	1970	[83,84]
		II*	4	ID/ ID+IM	25.5 µg/kg	w/o	96 burn pt.	Decreased mortality even in severe cases	1971	[85]
		II*	4+1	IM	3-12 µg/kg	w/o	361 oncologic pt.	Adverse events, reduced mortality	1973	[86]
		II*	6	IM	6-12 µg/kg	w/o	22 acute leukaemia pt. & 12 CF pt.	Adverse events in CF pt., no clinical benefit	1975	[87]
		II*	4+1	IM	0.17-0.68 mg	w/o	74 acute leukaemia children pt.	Increase in antibodies but a rapid decline. No clinical benefit	1973	[88]
	PEV-01, LPS from 16 PA serotypes	I	3	SC	0.5 mL	w/o	15 healthy indiv.	Variable antibody response	1976	[92]
		II	3	SC	0.5 mL	w/o	746 burn pt.	Reduced mortality, variable antibody response	1983	[91]

		II	3	SC	0.25 / 0.5 mL	w/o	34 CF pt.	No benefit in CF pt., no reduction in colonization and increased pt. deterioration	1984	[93]
	Aerugen®, LPS from 8 PA serotypes conjugated with ETA	I	2	SC	0.5 mL	w/o	20 healthy indiv.	Specific antibodies, safe	1987-1988	[95,98]
		II	3	IM	6-12 µg/kg	w/o	30 non-colonized CF pt.	Potential IgG specific response	1991-1997	[96,97]
		III	-	-	-	-	476 CF pt.	No clinical differences- stopped	2010	[99]
**Alginate**	2 types of mucoid exopolysaccharide obtained from mucoid PA	I	2	IM	10, 50, 100, 150 µg	w/o	28 healthy indiv.	Low opsonizing capacity, no improvement after booster	1994	[106]
**OMP**	OprF-OprI (IC43) systemic formulation	I	4	IM	20, 50, 100, 500 µg	$Al(OH)_3$	32 healthy indiv.	Opsonizing antibodies after 3rd dose	1999	[133]
		I	3	IM	100 µg	$Al(OH)_3$	8 burn pt.	Seroconversion in 7 pt.	2003	[134]
		I	2	IM	50, 100, 200 µg	$Al(OH)_3$	163 healthy indiv.	Specific IgG response	2014	[132]
		II	2	IM	100, 200 µg	$Al(OH)_3$	401 mechanically ventilated ICU pt.	Persistent specific IgG response	2017	[135]
		II/III	2	IM	100 µg	w/o	799 mechanically ventilated ICU pt.	No differences in survival or mortality rates, higher IgG titers	2020	[136]
	OprF-OprI (IC43) mucosal formulation	I	3	IN	500 µg	w/o	8 healthy indiv.	Higher IgG and IgA titers, safety	2001	[137]
	OprF-OprI (IC43), a combination of mucosal	I/II	2	IN/IM	1 mg/ 100 µg	$Al(OH)_3$	12 healthy indiv. (6 mucosal/ 6 mucosal	Well-tolerated and immunogenic, higher IgG	2003-2007	[138,139]

	and systemic formulations						and systemic booster)	levels with combining mucosal and systemic administration		
**Flagellin**	Monovalent flagellin	I	3	IM	40 µg	$Al(OH)_3$	220 healthy indiv.	Specific antibodies in serum and mucosa	1991	[198]
		II	3	IM	40 µg	w/o	10 healthy indiv.	Specific antibodies in serum and mucosa	1995	[197]
	Bivalent flagellin	III	4	IM	40 µg	$Al(OH)_3$ / thiomersal	483 non-colonized CF pt.	Delay in PA infection in CF pt. and increased survival rates	2007	[199]

CF, cystic fibrosis; ETA, exotoxin A; ICU, intense care unit; ID, intradermic route; Ig, immunoglobulin; IM, intramuscular route; IN, intranasal route; Indiv., individuals; LPS, lipopolysaccharide; OMP, outer membrane protein; PA, *P. aeruginosa*; PO, oral route; Pt., patient; w/o, without; SC, subcutaneous route.

***** Lack of regulation at the time
